# Association of adenoid hypertrophy and clinical parameters with preoperative polygraphy in pediatric patients undergoing adenoidectomy

**DOI:** 10.1007/s00405-024-09071-4

**Published:** 2024-11-20

**Authors:** Alexander Lein, Hasan Altumbabic, Miralem Đešević, Wolf-Dieter Baumgartner, Almir Salkic, Sekib Umihanic, Almedina Ramaš, Alen Harčinović, Andro Kosec, Faris F. Brkic

**Affiliations:** 1 Department of Otorhinolaryngology, Head and Neck Surgery, Medical University of Vienna, Waehringer Guertel 18-20, 1090 Vienna, Austria; 2 Department of Otorhinolaryngology, ASA Hospital, Sarajevo, Bosnia and Herzegovina; 3 PHI Eurofarm Center, Sarajevo, Bosnia and Herzegovina; 4 Department of Otorhinolaryngology, University Clinical Center Tuzla, Tuzla, Bosnia and Herzegovina; 5 Department of Otorhinolaryngology, Head and Neck Surgery, University Clinical Hospital Center Sestre Milosrdnice, Zagreb, Croatia

**Keywords:** Adenoidectomy, OSAS, Polygraphy, Pediatric, Clinical score

## Abstract

**Background:**

Adenotonsillar hypertrophy is the most frequent cause for obstructive sleep apnea (OSAS) in children. In patients with small tonsils and where adenoid size cannot be assessed, the indication for adenoidectomy often relies on clinical symptoms. However, data on the association of clinical parameters and adenoid hypertrophy with OSAS severity in children undergoing an adenoidectomy is sparse.

**Aim:**

To investigate the correlation of patient characteristics, adenoid hypertrophy, and clinical symptoms with OSAS severity in pediatric patients indicated for an adenoidectomy.

**Methods:**

We performed a retrospective chart review of all pediatric patients at our tertiary referral center between 2018 and 2023 who underwent polygraphy (PG) for OSAS diagnostics. Adenoid hypertrophy was assessed as adenoid-choanal ratio (AC-ratio) via nasal endoscopy and clinical symptom score (CS) via physical examination and parental survey. We included all symptomatic children with mild to severe OSAS (apnea–hypopnea index (AHI) ≥ 1). Exclusion criteria were obesity according to BMI and/or the presence of systemic diseases. The patients were divided according to age in a preschool and school cohort. Patient characteristics and PG data were compared between both groups. Linear regression analysis was used to investigate the association of AC-ratio, CS and BMI with the AHI.

**Results:**

A total of 121 patients were identified of which 81 were included in our study, resulting in 42 and 39 patients from 3–5 and 6–14 years of age, respectively. We observed a significant correlation between CS and BMI (*p* = 0.026) and the CS and AC-ratio (*p* < 0.001). Univariable regression analysis showed significant association of the AC-ratio and CS with AHI-score for the total (*p* < 0.001), the preschool (*p* < 0.001), and the school cohort (*p* < 0.001). In multivariable regression analysis, the significant association of AC-ratio and CS remained in the total (*p* = 0.014; *p* < 0.001), and the preschool cohort (*p* = 0.029; *p* = 0.002). However, only the CS remained as positive predictor in the school cohort.

**Conclusion:**

AC-ratio and clinical symptoms seem to be reliable predictors for OSAS severity in patients between 3–14 years of age. Moreover, only clinical symptoms were associated with OSAS severity in schoolchildren. Future investigation should contribute to the validation of our results

## Introduction

The obstructive sleep apnea syndrome (OSAS) is the most severe form of obstructive hypoventilation. It is characterized by repeated episodes of partial or complete upper airway collapse during sleep, leading to hypoventilation and abnormal sleep patterns [1, 2]. The prevalence of OSAS among children is estimated between 1 and 4% [2, 3]. Over time, OSAS can lead to a range of complications, including diminished sleep quality, heightened cardiovascular risk, persistent daytime fatigue, neuropsychological problems, and delayed neurocognitive development [4, 5].

The etiology of OSAS is multifactorial, with high body-mass-index (BMI), cranial abnormalities and different systemic diseases influencing its severity [6]. In pediatric patients, OSAS is frequently associated with relatively enlarged adenoids and tonsils, resulting from a growth mismatch where lymphoid tissue grows more rapidly than craniofacial structures [7]. This disproportion peaks between the age of 5 and 8 years [7, 8]. During sleep, the physiological loss of muscle tone increases the susceptibility to upper airway obstruction [9]. Consequently, adenotonsillectomy is recommended as the treatment of choice [10, 11].

In recent years, studies suggested that even pediatric patients with small tonsils may benefit from the combined resection of adenoids and tonsils, due to a resulting increase of pharyngeal patency during sleep [12]. However, there is a shift towards more individualized surgical strategies, addressing the unique anatomical and physiological needs of pediatric patients. Particularly tonsillotomy, has emerged as a viable alternative to tonsillectomy. However, in patients with small tonsils, adenoidectomy alone has shown promising results, effectively reducing OSAS symptoms without the need for tonsillar intervention [13, 14].

Despite Polysomnography being the gold standard for OSAS diagnostics, the choice for adenoidectomy relies on clinical findings and parental symptom reports [15]. Particularly, nasal endoscopy in children is associated with higher risk of fear and non-cooperative behavior, which negatively effects the quality of examination, prolongs consultations and increases distress [16]. A recent study showed that nasal endoscopy in children between 3 and 12 years of age can only be performed adequately in about 75% of cases [16]. Therefore, clinical symptoms play a crucial role in identifying OSAS and determining the need for adenoidectomy, particularly when tonsillar hypertrophy is absent on clinical examination and nasal endoscopy to confirm adenoid size is not feasible. However, data on the influence of clinical parameters and adenoid hypertrophy on OSAS severity in patients from 3–14 years of age are limited. Such data are urgently needed, as recent guidelines suggest that adenoidectomy alone may be sufficient for treating OSAS in children under two, supporting a shift towards less invasive and more conservative treatment approaches [13, 17].

In the present study, we aimed to determine whether patient characteristics, clinical parameters obtained through an easily obtainable symptom score, or the degree of nasal obstruction caused by adenoid hypertrophy serve as predictors of OSAS severity in children undergoing an adenoidectomy.

## Methods

### Patient and data collection

We included all pediatric patients referred to our tertiary referral center between 2018 and 2023 who presented with varying degrees of adenoid hypertrophy and without an indication for a tonsillectomy. Moreover, all included patients have undergone polygraphy (PG) preoperatively. Data was retrospectively analyzed. Exclusion criteria were the presence of obesity (BMI z-score > 2), genetic disorder including craniofacial abnormalities, cerebral palsy, neuromuscular disease, or any underlying systemic disease.

### Body-mass-index (BMI)

For age and development corrected BMI classification, the BMI z-score was calculated according to the WHO growth reference data (available at: https://www.who.int/tools/growth-reference-data-for-5to19-years/indicators/bmi-for-age and https://www.who.int/toolkits/child-growth-standards/standards/body-mass-index-for-age-bmi-for-age, accessed on May 1st, 2024). Patients were admitted to one of the following groups: underweight (BMI: < -1), normal (BMI: ≥ -1 and ≤ 1), overweight (BMI z-score > 1 and ≤ 2), and obese (BMI z-score: > 2).

### Nasal endoscopy and adenoid-choanal ratio (AC-ratio)

**Fig. 1:**
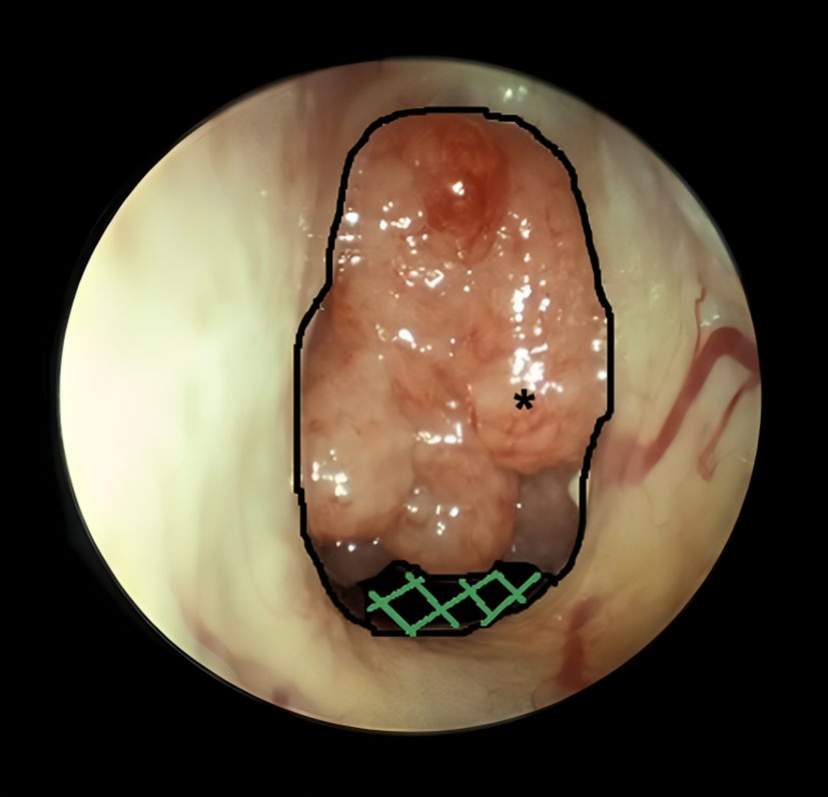
Endoscopic picture of a grade 3 obstruction. The black line marks the choana boundaries. The green checkered area marks the non-obstructed area. The remaining area is covered by adenoid tissue (*). Image used with kind permission of Hasan Altumbabic, MD

The endoscopic examination was conducted using a 2.7 mm rigid endoscope with a 0-degree angle (Storz, Tuttlingen, Germany) inserted through one nasal passage, providing a thorough visualization of anatomical structures relevant to our study. The data were saved in .mpg and VHS formats on both the hard disk and videotape. The images were digitally processed, and the adenoid size was calculated using the "Digimizer version 3.0.0.0" software. The adenoid size was measured as the percentage of adenoid tissue occluding the choanal area, referred to as the adenoid-choanal ratio (AC-ratio). Subsequently, the AC-ratio was assigned to one of three groups based on the degree of obstruction as previously described: grade 1 (0–33% obstruction), grade 2 (34–67% obstruction), and grade 3 (≥ 68% obstruction) [18]. Figure 1 shows an example of grade 3 obstruction.

### Clinical score (CS)

We collected clinical symptoms of patients through clinical examination and as observed by parents using a standardized questionnaire (Table 1). The assessed list of symptoms were (1) nasal obstruction, (2) snoring, (3) daytime mouth breathing, (4) disturbed sleep and/or daytime sleepiness, (5) sleep apnea observed by parents, and (6) hearing loss. During the clinical examination, the examiner asked the child's parent whether the symptom is present or not. This was answered with yes/present or no/not present and awarded 1 point for yes/present and 0 points for no/not present. Based on this information, a summary clinical score (CS) ranging from 0 to 6 was calculated for every patient.

**Table 1:** Questionnaire for standardized symptom assessment

Have you observed the following symptoms in your child?	
1. Nasal obstruction?	yes/no (1/0)
2. Snoring?	yes/no (1/0)
3. Daytime mouth breathing?	yes/no (1/0)
4. Disturbed sleep and/or daytime sleepiness?	yes/no (1/0)
5. Sleep apnea?	yes/no (1/0)
6. Hearing loss?	yes/no (1/0)
Summary score:	X/6

### Polygraphy (PG)

All PG data was assessed via the Philips® Alice NightOne Home Sleep Testing Device (Koninklijke Philips N.V., Amsterdam, The Netherlands, available at: https://www.philips.com/c-dam/b2c/master/experience/hs/sleep-better-live-better/ano-quickstart-guide.pdf accessed on 10th October, 2024) and analyzed via the Philips® Sleepware G3 with Somnolyzer software (Koninklijke Philips N.V., Amsterdam, The Netherlands, available at: https://www.philips.co.uk/healthcare/product/HC1082462/sleepware-g3-sleep-diagnostic-software, accessed on 5th August, 2024). PG records were subsequently evaluated independently using established scoring guidelines.^19^ Total sleep time (TST) was standardized for age according to the National Sleep Foundation's sleep time duration recommendation [20]. Sleep efficiency was calculated as follows: TST/time in bed*100%. Body position was measured as percentage of each position (left, supine, right and prone) of TST. Apnea was defined as cessation or reduction (> 90%) of oronasal airflow lasting for at least two respiratory cycles. Hypopnea was defined as a reduction of ≥ 50% in airflow accompanied by oxygen desaturation (SpO2) of ≥ 3%, or with arousal, lasting for two respiratory cycles. The AHI was defined as the average number of apnea and hypopnea per hour. Consequently, patients were admitted to one of the following OSAS severity groups according to clinical guidelines^21^: mild (AHI: ≥ 1 and ≤ 4), moderate (AHI: > 4 and < 10) and severe (AHI: ≥ 10) OSAS. Body position was measured as percentage of every position of total sleep time.

### Statistical analysis

The data collected was compiled and structured using a Microsoft Excel spreadsheet (Microsoft Corporation, 2018. Microsoft Excel, Available at: https://office.microsoft.com/excel). To test for normality the Shapiro–Wilk test was used. For difference between continuous variables the ANOVA and Kruskal–Wallis test were used for parametric and non-parametric variables, respectively. The chi-square test was used to test for significance between categorial variables. If one group was below five, the Fischer’s-exact test was used. Correlation between variables was calculated with Pearson’s correlation. Univariable and multivariable linear regression was used to predict AHI via different variables. A p-value of < 0.05 was considered significant.

### Ethical considerations

The study adhered to ethical guidelines outlined in the Declaration of Helsinki. Patient confidentiality was strictly maintained, and the data were anonymized for analysis. Due to retrospective nature of the study, the informed consent was waived.

## Results

### Patient characteristics

In total, we identified 121 patients. Given that obesity is a prevalent risk factor for OSAS [6], and to ensure the validity of our findings, we excluded obese patients from our analysis. Consequently, 40 patients were excluded due to a BMI z-score > 2. Finally, 81 patients were included in our study, with ages ranging from 3 to 14 years. All assessed patient characteristics are shown in Table 2. The patients were stratified by age as previously described [7], resulting in two cohorts: a preschool cohort (< 6 years, n = 42) and a school cohort (≥ 6 years, n = 39). The mean ages for the preschool and school cohorts were 4.1 and 8.3 years, respectively.

**Table 2:** Patient characteristics of the total, preschool, and school cohort

Variable		Total Mean/n [± SD/%]	Preschool Mean/n [± SD/%]		School Mean/n [± SD/%]	p-value
Patient number		81(100)	42 (100)		39(100)	
Age		6.12 (2.7)	4.17 (0.8)		8.33 (2.2)	** < 0.001**
Gender	female	47 (58.0)	26 (61.9)		21 (53.8)	
	male	34 (42.0)	16 (38.1)		18 (46.2)	0.463
BMI		15.90 (2.0)	15.59 (1.8)		16.24 (2.2)	0.162
BMI z-score		0.01 (1.1)	0.05 (1.0)		-0.03 (1.2)	0.993
Weight						
	normal	66 (81.5)	34 (81.0)		32 (82.1)	
	overweight	15(18.5)	8 (19.0)		7 (17.9)	0.899
AC-ratio						
	1	2 (2.5)	1 (2.4)		1(2.6)	
	2	30(37.0)	13 (31.0)		17 (43.6)	
	3	49(60.5)	28 (66.7)		21 (53.8)	0.575
CS						
	3	4(4.9)	3 (7.1)		1(2.6)	
	4	41 (50.6)	16 (38.1)		25 (64.1)	
	5	24(29.6)	15 (35.7)		9(23.1)	
	6	12 (14.8)	8 (19.0)		4(10.3)	0.130

*AC-ratio* adenoid-choanal ratio, *BMI* body mass index, *CS* clinical score, *SD* standard deviation

Statistically significant results are depicted in bold print

There was no significant difference in BMI z-score between both groups (*p* = 0.993). The weight distribution of normal weight (81.0% vs. 82.1%) and overweight (19.0% vs. 17.9%) patients did not differ significantly between the preschool and school cohorts (*p* = 0.899).

In total, the AC-ratio was categorized as grade 3 in 49 patients (60.5%), grade 2 in 30 patients (37.0%), and grade 1 in 2 patients (2.5%). There was no significant difference in the distribution of AC-ratio between the two cohorts (*p* = 0.575). The clinical symptom burden, measured by CS, ranged from a minimum of 3 (n = 4) to a maximum of 6 (n = 12). In the preschool group, a higher proportion of patients had a CS of 3 (7.1% vs. 2.6%), 5 (35.7% vs. 23.1%), and 6 (19% vs. 10.3%). Conversely, the school cohort had a higher proportion of patients with a CS of 4 (64% vs. 38.1%). However, there was no significant difference in the distribution of CSs between the two groups (*p* = 0.130).

### PG

All assessed PG variables are shown in Table 3. Overall, all children showed good sleep quality, with a sleep efficiency of 98.4% (± 1.8%). Total sleep time ranged from 4.7 to 11.1 h, with a mean of 7.52 h (± 1.47 h) for the preschool group and 7.52 h (± 1.23 h) for the school group (*p* = 0.580). There was no significant difference in AHI between the preschool and school cohorts, with 12.9 (± 8.6) events/h and 11.4 (± 8.6) events/h, respectively (*p* = 0.357). Additionally, there were no significant differences in apnea (*p* = 0.508) and hypopnea (*p* = 0.095) events between both groups.

**Table 3:** Polysomnography data variables by age group

	Total Mean/n (± SD/%)	Preschool Mean/n [± SD/%]	School Mean/n [± SD/%]	p-value
Variable				
Total sleep time [min]	451.0 (81.1)	451.0 (88.1)	451.1 (74.0)	0.580
Sleep efficiency [%]	98.4 (1.8)	98.4(1.7)	98.4 (1.9)	0.640
AHI [events/h]	12.2 (8.6)	12.9(8.6)	11.4 (8.6)	0.357
AHI grouped				
mild	5.0(6.2)	3.0(7.1)	2.0(5.1)	
moderate	39.0(48.2)	17.0(40.5)	22.0(56.4)	
severe	37.0(45.7)	22.0(52.3)	15.0(38.5)	0.338
Total apnea events	54.3 (44.8)	57.6(46.7)	50.8 (43.0)	0.508
Total hypopnea events	22.2 (20.3)	25.7(23.0)	18.3 (16.4)	0.095
Average apnea duration				
average (sec)	20.1(7.0)	21.1(7.6)	19.0(6.2)	0.277
sum (min)	40.0(30.4)	43.3(32.3)	36.4(28.0)	0.343
Lowest Sp02 [%]	79.2 (8.0)	77.3(7.2)	81.2 (8.5)	**0.011**
Heart rate [bpm]				
lowest	51.0(12.0)	49.7(13.5)	52.4(10.2)	0.441
average	84.4(11.6)	90.2(10.0)	78.1(9.7)	** < 0.001**
highest	166.9(56.1)	183.2(50.2)	149.4(57.4)	**0.003**
Total snoring time [min]	33.4 (44.4)	32.0(39.7)	34.8 (49.4)	0.467
Body position [% of TST]				
left	20.5(14.2)	15.0(12.0)	26.5(14.1)	** < 0.001**
supine	56.3(20.0)	62.4(19.7)	49.8(18.4)	**0.005**
right	19.6(14.7)	19.9(15.1)	19.3(14.5)	0.843
prone	3.5(5.6)	2.7(5.6)	4.4(5.6)	0.144

*SpO2* oxygen saturation measured by oximetry, *AHI* apnea–hypopnea index, *TST* total sleep time, *sec* seconds, *min* minutes, *bpm* beats per min, *h* hours

Statistically significant results are depicted in bold print

A significant difference was observed in the lowest measured oxygen saturation, with the preschool group having 77.3% (± 7.2%) SpO2 compared to 81.2% (± 8.5%) SpO2 in the school group (*p* = 0.011).

The preschool group exhibited higher average heart rates (90.2 bpm vs. 78.1 bpm; *p* < 0.001) and higher peak heart rates (183.2 bpm vs. 149.4 bpm; *p* = 0.003). However, there was no significant difference in the lowest heart rates between the groups (49.7 bpm vs. 52.4 bpm; *p* = 0.441).

Total snoring times were comparable between the preschool and school cohorts, with 33.4 min and 34.8 min, respectively (*p* = 0.467). In terms of body position during sleep, preschool children spent significantly less time sleeping on their left side (15% vs. 26.5%; *p* < 0.001) and more time in the supine position (62.4% vs. 49.8%; *p* = 0.005).

### Correlation

Next, we investigated the correlations between the AC-ratio, CS, and BMI z-score for the entire cohort, as shown in Table 4. We observed a positive correlation between the CS and the AC-ratio (r = 0.633; *p* < 0.001), as well as between the CS and the BMI z-score (r = 0.202; *p* = 0.026). However, there was no significant correlation between the AC-ratio and the BMI z-score (r = 0.118; *p* = 0.198).

### Regression analysis

**Fig. 2:**
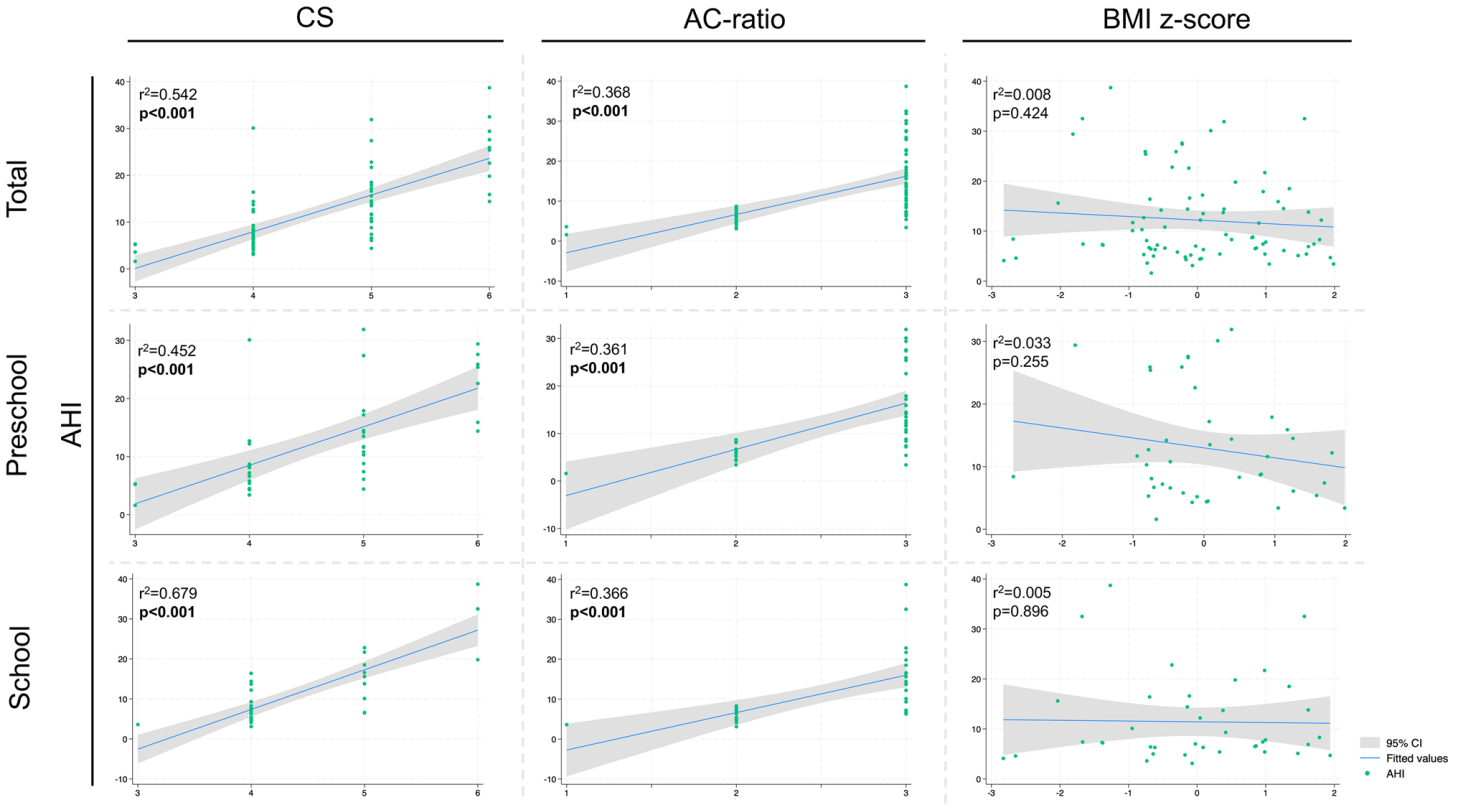
Scatter plot of linear regression of AC-ratio, CS and BMI z-score with AHI for the whole, preschool and school cohort. Statistically significant results are depicted in bold print. Abbreviations: AC-ratio, adenoid-choanal ratio; AHI, apnea–hypopnea ratio; BMI, body-mass-index; CI, confidence interval; CS, clinical score

Finally, we analyzed the prediction of AHI severity using the AC-ratio, CS and BMI z-score across all cohorts. The results of the linear regression are shown in Fig. 2.

Univariable analysis revealed that both the AC-ratio (HR: 6.78; 95%CI: 6.76–12.38; *p* < 0.001) and the CS (HR: 9.67; 95%CI: 6.23–9.45; *p* < 0.001) were associated with AHI severity in the total cohort (Table 5). These findings were consistent in the preschool cohort for both the AC-ratio (HR: 4.75; 95%CI: 5.59–13.86; *p* < 0.001) and the CS (HR: 5.75; 95%CI: 4.3–8.97; *p* < 0.001), as well as in the school cohort (AC-ratio: HR: 4.62; 95%CI: 5.26–13.47; *p* < 0.001 and CS: HR: 8.86; 95%CI: 7.65–12.18; *p* < 0.001). However, the BMI z-score did not reach significance in predicting AHI severity in the whole cohort (*p* = 0.424), the preschool cohort (*p *= 0.255), or the school cohort (*p* = 0.896).

**Table 4:** Correlation matrix of AC-ratio, CS and BMI z-score

	AC-ratio	CS	BMI z-score
AC-ratio	1		
CS	0.633	1	
	**(p < 0.001)**		
BMI z-score	0.118	0.202	1
	(p = 0.198)	**(p = 0.026)**	

*AC-ratio* adenoid-choanal ratio, *BMI* body-mass-index, *CS* clinical score

Statistically significant results are depicted in bold print

In the multivariable analysis, the AC-ratio (HR: 2.53; 95%CI: 0.8–6.79; *p* = 0.014) and the CS (HR: 6.16; 95%CI: 4.22–8.26; *p* < 0.001) remained significant positive predictors of AHI severity for the whole cohort (Table 5). This was also observed in the preschool cohort for both the AC-ratio (HR: 2.27; 95%CI: 0.56–9.83; *p* = 0.029) and the CS (HR: 3.33; 95%CI: 1.82–7.46; *p* = 0.002). However, in the school cohort, only the CS remained significant in the multivariable analysis (HR: 6.07; 95%CI: 5.98–11.99; *p* < 0.001).

## Discussion

Over the past decades, adenoidectomy with or without tonsillectomy has been the standard treatment for children with OSAS. However, this procedure is associated with significant adverse effects. Consequently, there is a growing trend towards more selective patient criteria to determine who should undergo this surgery. In the present study, we could show, that adenoid size, as measured by choanal obstruction, is a positive predictor of AHI severity in children from 3–14 years of age with small tonsils. Furthermore, clinical symptoms seem to be a stronger predictor, especially in children between 6 and 14 years of age. Therefore, our study adds to the growing knowledge on the association of clinical variables in specific OSAS patient groups, paving the way for personalized therapy approaches.

The relationship between lymphatic tissue size and the severity of OSAS has been a topic of ongoing debate. In 1998, Brooks et al. showed a significant association between adenoid size and apnea duration, and adenoid size and lowest SpO2 saturation [22]. However, adenoid and tonsil size did not predict total number of apneas [22]. In contrast, Hwang et al. found a positive correlation of adenoid size with AHI severity (rho = 0.293; *p* = 0.022) [15]. The authors emphasize that this correlation might be of no clinical significance. Nevertheless, we found that the AC-ratio served as an independent predictor of AHI only in children aged 3–6 years, but not in those aged 7–14 years. These findings support an age-dependent relationship between adenoid size and nasopharyngeal space. Yan et al. noted that the nasopharyngeal space is narrowest at 4.5 years, while adenoid size peaks at 7–10 years [23]. Similarly, Tagaya et al. observed a significant association of AHI in preschool but not in school-aged children [24]. Recent cephalometric studies confirm this age-dependent relationship, emphasizing the importance of considering these age groups separately [7, 25]. Moreover, this may explain why the AC-ratio was not a predictor for children aged 6–14 in our multivariable analysis.

Given the difficulty in measuring adenoid size clinically, variability in measurement methods likely contribute to differing observations regarding adenoid size influence on OSAS severity. Studies by Brooks et al. [22] and Yan et al. [26]. used radiographic methods proposed by Fujoka et al. [27], which may not reflect the anatomical conditions relevant for OSAS development. Parik et al. used an endoscopic approach, grading adenoid hypertrophy based on its anatomical relationship to surrounding structures [28]. In line with our results, subsequent follow-up studies have shown a strong correlation between this method and AHI score [24, 29]. Therefore, assessing adenoid size in relation to nasal obstruction, might better reflect the anatomical important relationships for predicting OSAS severity.

One important strength of the present study is the analysis of the specific impact of adenoid hypertrophy on OSAS severity. Certainly, the relative influence of tonsils versus adenoids remains a topic of ongoing debate. A recent meta-analysis showed that tonsillar hypertrophy (RR = 3.55) may be the leading risk factor for the obstructive sleep apnea, even before family history (RR = 3.03) or adenoid hypertrophy (RR = 1.63) [6]. In contrast, a study by Kaneko et al. found higher values for adenoid hypertrophy (RR = 10.4) than tonsil hypertrophy (RR = 4.42) [30]. The importance of distinguishing between patients with adenoid or tonsil hypertrophy with adenoidectomy and/or tonsillectomy was shown by Domany et al. The authors reported on 500 children either receiving adenotonsillectomy or adenoidectomy with different grades of tonsil hypertrophy [31]. While the authors observed no difference between both interventions, interestingly, in the adenoidectomy group, 30% of patients had grade + 3 tonsils, and had worse postoperative outcomes [31]. In this group, adenoidectomy was in any case related with higher values of persisting OSAS symptoms (20% vs. 9.8%) [31].

In current guidelines, snoring is highlighted as the primary clinical symptom prompting OSAS diagnostic evaluation in pediatric patients.^1^ Our study demonstrated a strong correlation between our clinical symptoms score and AHI severity in children aged 3–5 and 6–14 years (*p* = 0.002, *p* < 0.001). Over the past decade, several clinical questionnaires, such as the Pediatric Sleep Questionnaire by Chervin et al. [32] and the Clinical Severity Scale by Spruyt et al. [33], have been developed to predict OSAS severity on symptom evaluation. However, questionnaires are timely intensive and make their routine use in clinical practice challenging. In the present study, the binary scoring system was chosen to streamline the assessment process and to ensure quick and consistent symptom evaluation, which is essential for practical use in a busy clinical environment. Symptom based clinical scoring systems, similar to the Centor score [34] used for assessing bacterial tonsillitis, hold significant clinical value. Therefore our results could pave the way for a streamlined, symptom-based approach to evaluate whether adenoidectomy is necessary. Future studies with larger cohorts are needed to validate our results and further elucidate the predictive ability of clinical symptoms on OSAS severity in patients between 3–14 years of age.

In this study, we identified adenoid size as an independent predictor of OSAS severity in children under 6 years with small tonsils. The optimal treatment strategy for OSAS is under ongoing debate, with a growing trend towards individualized, conservative approaches. For patients with obesity, lifestyle modifications, particularly weight reduction, should be the primary intervention [35]. Regarding surgical intervention, a meta-analysis from 2015 found that tonsillotomy is comparable to tonsillectomy in resolving airway obstruction and improving quality of life, while offering advantages of lower hemorrhage risk, shorter procedure times, and faster recovery [36]. While being superior in the early post-operative phase tonsillotomy seems to have lower long term stability in alleviating upper airway obstruction [37, 38]. In the context of our study on children with smaller tonsils, pharmacological interventions should also be considered [38]. Especially, nasal steroids can reduce adenoid size through anti-inflammatory effects and potentially eliminate the need for surgery. In a randomized controlled trial by Tapia et al., intra-nasal fluticasone propionate use twice-daily reduced the AHI score from 3.9 to 1.9 over three months in children aged 5–12 [38, 39]. Another study demonstrated that a 6-week nasal budesonide regimen reduced both adenoid size and AHI from 3.7 to 1.3 in children with mild OSAS [38, 40]. Notably, a Cochrane systematic review did state insufficient evidence for the efficacy of intranasal corticosteroid application for OSA treatment [41]. However, studies mainly focused on children older than 5 years. Considering our findings, younger children with isolated adenoid hypertrophy, particularly those under 5 years, may be ideal candidates for pharmacological treatment. Future studies should explore conservative and personalized therapeutic approaches in this age group, potentially reducing the need for surgical intervention.

### Limitations

The present study has several limitations. First, the retrospective nature of our study comes with inherent limitations like limited data availability, which did not allow us to stratify the exact symptoms comprising the clinical score. Secondly, we assessed PG with a wearable diagnostic home sleep testing platform, which may introduce bias due to patient handling issues. However, the platform is well established and used as reference for new home sleep testing platforms [42]. Moreover, data on postoperative PG were not available, thus emphasizing future studies regarding adenoidectomy and its influence on PG variables in this age group. For calculation of BMI z-score we used the WHO standards. However, the age- and sex-specific reference is different for every country. Therefore, our study only refers to patients specific for our region. While the cohort in our study is relatively low, bigger studies are needed to confirm our results Table 5.

**Table 5:** Univariable and multivariable linear regression analysis

	Univariable					Multivariable			
	HR	95%CI		R^2^	p-value	HR	95%CI		p-value
Total									
AC-ratio	6.78	(6.76 −	12.38)	0.368	** < 0.001**	2.53	(0.80 −	6.79)	**0.014**
CS	9.67	(6.23 −	9.45)	0.542	** < 0.001**	6.16	(4.22 −	8.26)	** < 0.001**
BMI z-score	− 0.8	(− 2.44 −	1.04)	0.008	0.424	− 1.69	(− 2.1 −	0.17)	0.096
Preschool									
AC-ratio	4.75	(5.59 −	13.86)	0.361	** < 0.001**	2.27	(0.56 −	9.83)	**0.029**
CS	5.75	(4.30 −	8.97)	0.452	** < 0.001**	3.33	(1.82 −	7.46)	**0.002**
BMI z-score	− 1.16	(− 4.36 −	1.19)	0.033	0.255	− 1.83	(− 3.75 −	0.18)	0.074
School									
AC-ratio	4.62	(5.26 −	13.47)	0.366	** < 0.001**	1.01	(− 1.94 −	5.80)	0.318
CS	8.86	(7.65 −	12.18)	0.679	** < 0.001**	6.07	(5.98 −	11.99)	** < 0.001**
BMI z-score	− 0.13	(− 2.47 −	2.17)	0.005	0.896	− 0.73	(− 1.8 −	0.85)	0.468

*AC-ratio* adenoid-choanal ratio, *AHI* apnea–hypopnea ratio, *BMI* body-mass-index, *CI* confidence interval, *CS* clinical score

Statistically significant results are depicted in bold print

## Conclusion

The AC-ratio and our CS score seem to be viable AHI prognosticators in children with OSAS and small tonsils. The easily obtainable score makes it highly suitable for routine clinical practice. Regarding the ongoing trend to more tailored surgical approaches, our study could guide future studies addressing the unique anatomical and physiological needs of pediatric patients with OSAS. Future investigations should contribute to the validation of our results in larger cohorts. Studies including postoperative AHI assessment could explore specific cut-offs for the CS score that may inform surgical intervention thresholds. Moreover, these studies may investigate pharmacological treatment options in this specific patient cohort.

## Data Availability

Data are available from the corresponding author upon reasonable request**.**
